# Radiation-Induced Immune Responses from the Tumor Microenvironment to Systemic Immunity

**DOI:** 10.3390/cancers17233849

**Published:** 2025-11-30

**Authors:** Shaun Png, Sirimuvva Tadepalli, Edward E. Graves

**Affiliations:** 1 Department of Radiation Oncology, Stanford University, Stanford, CA 94305, USA; shaunpng@stanford.edu; 2 Department of Microbiology and Immunology, Indiana University School of Medicine, Indianapolis, IN 46202, USA

**Keywords:** radiotherapy, immunology, tumor microenvironment, abscopal effect, systemic radiation response, normal tissue irradiation’ immune modulation

## Abstract

Radiotherapy remains a central component of cancer therapy that induces DNA damage and cancer cell death. Beyond its cytotoxic effects, radiotherapy acts as a potent immunomodulator by releasing immunogenic molecules, inflammatory mediators, and neoantigens that shape anti-tumor immunity. Radiation-induced immune responses can have opposing effects, including immune activation or immunosuppression that dictate local tumor responses. Increasing evidence suggests these immunologic effects are not confined to the site of irradiation, as radiation exposure to surrounding normal tissue can trigger systemic immune signaling that affects local tumor progression as well as metastatic spread. This review examines radiotherapy-induced immune responses across three interconnected contexts: (i) tumor-intrinsic signaling during radiation; (ii) tumor microenvironmental changes where innate and adaptive immune responses alter local outcomes; and (iii) systemic and off-target effects contributing to broader immune remodeling. A comprehensive understanding of radiotherapy-induced immune responses will guide therapeutic strategies to enhance its immunological potential while minimizing unintended immune and off-target effects.

## Simple Summary

Radiotherapy is a cornerstone of cancer treatment that eliminates tumor cells by damaging their DNA. However, radiation also profoundly influences the immune system, activating or suppressing immune responses that can inhibit or promote tumor growth, respectively. These effects extend beyond the treated tumor, as irradiation of adjacent normal tissues can trigger immune responses throughout the body. This review summarizes how radiation shapes tumor immunity, alters immune cell recruitment and function in surrounding normal tissues, and drives systemic effects that influence cancer progression and metastasis. An understanding of these mechanisms will help harness radiotherapy to strengthen anti-tumor immunity while minimizing off-target immune complications.

## 1. Introduction

While the classical dogma of radiobiology attributes the therapeutic effect of radiotherapy to cytocidal DNA damage and tumor cell death, the role of the immune system in modulating the therapeutic response has gained increasing attention recently [1]. The immune system can be broadly characterized into two complementary components: the innate and adaptive immune system. The innate immune response acts rapidly in a non–antigen-specific manner, serving as the “first line of defense” and the earliest responder to inflammatory **stimuli (i.e., radiation).** In contrast, the adaptive immune response develops more slowly in an antigen-specific manner. An effective immune response relies on the coordinated interplay between both arms to generate and regulate an appropriate response to the inflammatory stimuli. Following radiation damage and cell death, damage-associated molecular patterns (DAMPs) permeate the tumor microenvironment (TME) and are sensed by the immune system. This leads to the activation of multiple downstream signaling pathways culminating in the release of cytokines and chemokines (discussed in Section 2). Consequently, innate immune cells are recruited to the TME to mediate effector or regulatory functions (discussed in Section 3). Radiation-induced immune activation can act as an in situ vaccine with the potential to activate antitumor T cells and generate an anti-tumor immune response. Conversely, immune signals released by radiation can promote the recruitment of immunosuppressive myeloid and regulatory T cells that aid tissue repair and suppress adaptive immunity [2,3]. Amidst the ongoing debate of radiotherapy being inherently immunostimulatory or immunosuppressive, it is crucial to understand that the adaptive immune response (discussed in Section 4) significantly modulates the therapeutic efficacy of radiotherapy. Although radiotherapy-induced immune modulation has been extensively investigated in the context of local tumor irradiation, much less is known about the systemic response that may accompany treatment. Most existing work has focused on localized effects or on the abscopal phenomenon, where irradiation of one tumor site elicits immune-mediated regression at distant sites. However, radiation exposure is often not confined solely to the tumor tissue, and normal tissue within the field or periphery of treatment may experience off-target irradiation. Crucially, such effects may have implications on immune infiltration in the TME (discussed in Section 5). Given the diverse and often opposing roles that immune cells play within the TME, it is critical to elucidate how specific immune cell populations influence the tumor’s response to radiotherapy, as these interactions can either potentiate treatment efficacy or contribute to therapeutic resistance.

## 2. Radiotherapy-Induced Immune Signals from Tumor Cells

**Figure 1:**
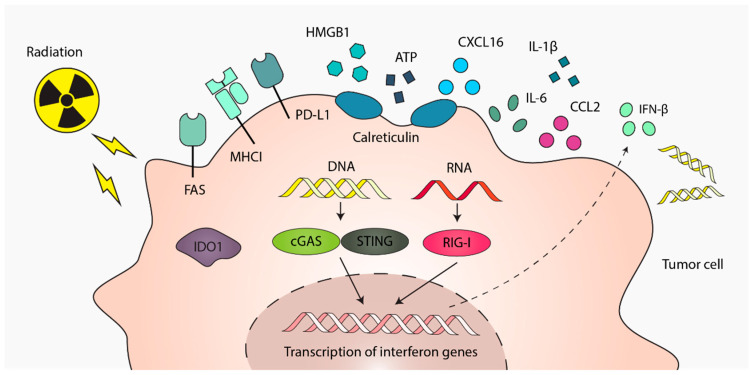
Radiation induces expression and release of factors that drive immunogenic cell death (ICD), as well as cytokines and chemokines that promote immune cell recruitment and regulate their function. IFN-β production is a key regulator of radiation-induced immune responses triggered by cytoplasmic DNA/RNA accumulation. Radiation also upregulates certain cell-surface receptors that engage immune cells and metabolic enzymes such as indoleamine 2,3-dioxygenase 1 (IDO1) that modulate immune activity.

Radiotherapy influences a myriad of tumor-derived signals that influence the downstream immune response (Figure 1). In the early timepoints following irradiation, DNA and RNA molecules accumulate in the cytoplasm due to the loss of nuclear structural integrity, leading to the translocation of nucleic acids from the nucleus to the cytoplasm. DNA accumulation in the cytoplasm can be observed as early as 24–48 h after irradiation and is accompanied by increased translocation of proteins involved in DNA-sensing pathways [4]. Double-stranded DNA (dsDNA) within tumor cells is recognized by DNA damage sensors, activating stimulator of interferon genes (STING)-dependent production of type 1 interferon (IFN-I) [5]. Additionally, endogenous RNA accumulation in the cytoplasm can be detected by retinoic acid-inducible gene I (RIG-1), further promoting IFN-I production by tumor cells [6]. More recently, the mTOR-LTR-RIG-1 axis has been shown to regulate IFN-I in cancer tissues, which is implicated in the recruitment of macrophages and dendritic cells (DCs) to the TME following radiation [7]. In response to irradiation, tumor cells also upregulate several cell surface receptors that mediate interactions with immune cells, ultimately shaping either pro- or anti-tumor immune responses. For instance, major histocompatibility Complex I (MHCI), programmed death-ligand 1 (PD-L1), and Fas cell surface death receptor (FAS) are recognized by T-cells [8,9,10]. While recognition of MHCI and FAS by T-cells promotes anti-tumor T-cell responses, engagement of PD-L1 can suppress T-cell responses. Additionally, indoleamine 2,3-dioxygenase 1 (IDO) expression in tumor cells facilitates the recruitment and activation of immunosuppressive myeloid-derived suppressor cells (MDSCs) to inhibit anti-tumor immunity [11].

The induction of DNA damage and cellular stress following radiation leads to the release of various immune mediators and DAMPS that facilitate immunogenic cell death (ICD). Nucleic acids are released upon cell death and can be picked up by immune cells, triggering various signaling pathways that propagate and amplify inflammatory responses. Of note, tumor-derived dsDNA can be captured by phagocytic DCs, triggering STING-dependent production of IFN-I in the TME [12]. Tumor cells also release adenosine triphosphate (ATP) [13], high mobility group box 1 protein (HMGB1) [13,14], and calreticulin [13,15], which are recognized by cell surface receptors P2X7, CD91, and toll-like receptor 4 (TLR4), respectively, on DCs. ATP-P2X7 interactions activate NOD-, LRR- and pyrin domain-containing protein 3 (NLRP3) inflammasome-dependent release of IL-1b, the absence of which inhibits adaptive immunity [16]. In addition, calreticulin-CD91 and HMBG1-TLR4 signaling facilitate antigen processing by DCs and presentation of tumor antigens to T-cells [17]. In addition, the release of tumor neoantigens following radiation-induced cell death can further prime DCs to activate T-cells [8]. Therefore, tumor-derived DAMP signals and neoantigens picked up by DCs play a key role in facilitating anti-tumor T-cells responses.

The cytokine and chemokine microenvironment induced by irradiation also play a role in the recruitment of immune cells to the TME. Following irradiation, tumors upregulate genes involved in the recruitment of immune cells, releasing cytokines and chemokines such as IL-6, CCL-2, IL-1b to facilitate immune cell trafficking to the TME [18]. Some tumors have also been shown to release CXCL16 upon radiation to directly recruit T-cells to the TME [19]. Once in the TME, infiltrating immune cells can further shape the inflammatory environment by secreting their own proinflammatory cytokines or chemokines, recruiting additional immune cells [17]. We further review the cytokines regulating immune cell recruitment and function in Section 3 and Section 4.

The composition of released factors and the inflammatory milieu shortly after irradiation are broadly influenced by two key factors: the radiation dose regimen and the intrinsic characteristics of the tumor itself. For example, high radiation doses can activate the exonuclease Trex1, leading to DNA degradation and reduced tumor immunogenicity [20]. Moreover, dose-dependent release of the immunogenic cell death mediators ATP and HMGB1 has been demonstrated, which could affect the magnitude of DC activation and subsequent activation of the adaptive immune system [13]. In addition, different tumor types have been shown to exhibit distinct gene expression and cytokine profiles following irradiation, which correlate with the tumor’s immunogenicity and the extent of immune infiltration in response to radiation [21]. Crucially, local surrounding non-tumor tissue within the irradiated clinical volume is also susceptible to radiation-induced cell death and can release DAMPs and molecular mediators to shape the inflammatory milieu of the TME [22]. Of note, radiation-induced damage to endothelial cells can facilitate the recruitment of inflammatory immune cells to protect normal tissues [23]. However, the differential contribution of tumor-derived and normal-tissue-derived DAMPs within the TME remain understudied, and further work is needed to understand their distinct roles in regulating the inflammatory milieu after radiation.

## 3. The Innate Immune Response to Radiotherapy

The upregulation of numerous inflammatory cytokines and chemokines within the TME after radiotherapy facilitates the recruitment of immune cells [18]. Neutrophils are among the first immune cells to respond to radiation exposure in both tumors and normal tissues, as soon as 12–24 h after irradiation [24]. Tumor-derived IL-1β facilitates the upregulation of CXCLs, which can recruit neutrophils in a CXCR2-dependent manner [25]. Circulating monocytes are also among the primary responders to radiation with the ability to differentiate into monocyte-derived cells with distinct phenotypes and functions that can influence the efficacy of radiotherapy [26,27,28]. Depending on the phenotype, monocyte-derived cells can be recruited to the TME by chemokines CCL-2 and CCL-5 [18,27]. Among monocyte-derived cells, MDSCs have been shown to be recruited through the colony stimulating factor 1 (CSF1)/CSF1R signaling axis [29] in a CCL-2 dependent manner [30,31], which is compromised in STING-deficient mice [30]. Additionally, hypoxia-inducible factors (HIF) released in response to radiation further facilitate the recruitment of MDSCs [32,33]. Radiation also stimulates the release of CCL11, which has been shown to recruit anti-tumor eosinophils into the TME [34]. Natural killer (NK) cells are cytotoxic innate immune effector cells capable of directly killing tumor cells, and their recruitment via CXCL18 following radiotherapy has been shown to be critical for mediating anti-tumor responses in certain models [35]. In addition to their direct cytotoxic function, NK cells can act as a bridge to adaptive immunity by promoting DC activation [36]. Given their potential, combining NK cell-based approaches with radiotherapy represents a promising strategy to enhance anti-tumor effects of radiotherapy [37,38,39].

A key mediator regulating the anti-tumor function of innate immune cells in the TME is IFN-I. As previously discussed, the cyclic GMP-AMP synthase (cGAS)-STING-dependent cytosolic DNA sensing pathway in DCs has been demonstrated to be crucial for IFN-I induction after irradiation [12,40]. IFN-I regulates the cross-presentation of antigens by cDC1s to CD8^+^ T-cells, which is critical for activating CD8^+^ cells and the adaptive anti-tumor response [41]. IFN-I is also critical for the activation of monocytes in an autocrine manner that is essential for CD8^+^ T cell function [27]. IFN-I has also been associated with NK cell recruitment following radiotherapy in combination with Ataxia-Telangiectasia Mutated and Rad3-Related (ATR) inhibitors, further mediated by tumor-derived CXCL8 [42]. Therefore, IFN-I appears to be a key mediator in regulating the anti-tumor function of innate immune responses following radiotherapy.

However, immune cells recruited to the TME can also exert immunosuppressive functions. As briefly discussed above, MDSCs are known for their immunosuppressive properties and can suppress CD8^+^ T-cell responses [1]. Of note, while MDSCs are commonly associated with an immature myeloid progenitor population, the markers used to identify these cells (F4/80 and Gr1) are also expressed by mature monocytic and granulocytic subsets [43], which could lead to the inadvertent inclusion of other immune cells as MDSCs in response to radiation. Nevertheless, MDSC function is supported by transforming growth factor β (TGF-β) and the hypoxic environment, both of which are increased following radiation exposure [32,33]. TGF-β also plays a key role in regulating the immunosuppressive function of neutrophils and macrophages. TGF-β can also support the wound-healing and regulatory function of macrophages, which further support the immunosuppressive TME by producing cytokines associated with angiogenesis and wound healing [44]. These macrophages further contribute to an immunosuppressive environment by producing more TGF-β [45]. Similarly, TGF-β also polarizes neutrophils to a pro-tumor phenotype [46]. In response to radiation, neutrophils can be activated to produce iDO1 and arginase, which are thought to inhibit T-cell responses [24]. Given the central role of TGF-β in supporting the immunosuppressive function of immune cells, depletion of TGF-β in combination with radiotherapy has been observed to significantly enhance the therapeutic efficacy of radiotherapy [47].

Specific targeting of these immunosuppressive cells can better promote anti-tumor responses. For instance, depletion of MDSCs via anti-CCL2 treatment can enhance the therapeutic efficacy of radiotherapy [30,31]. The anti-tumor function of macrophages can be activated by low dose-radiotherapy [48] or through the removal of the “don’t eat me” signal CD47 on tumor cells, which can be recognized by the receptor signal regulatory protein α (SIRPα) on macrophages [49]. CXCL2-dependent recruitment of neutrophils has been shown to support anti-tumor responses in some instances [25,50], and can be enhanced when combined with G-CSF treatment [50]. While monocytes may serve as a reservoir to generate immunosuppressive MDSCs, they themselves appear to potentiate anti-tumor response by promoting a CD8^+^ T-cell response. Interestingly, this phenomenon occurs as a result of the off-target irradiation of normal tissue [27]. Therefore, methods to preferentially activate the anti-tumor function of monocytes with radiotherapy could serve to improve anti-tumor response. However, the differential contributions of irradiated tumor and normal tissues in the recruitment of innate immune cells remain understudied, and further work is needed to understand their role especially in cases of deep-seated tumors where exposure to normal tissue during radiotherapy is unavoidable.

## 4. The Adaptive Immune Response to Radiotherapy

Radiotherapy has the potential to direct adaptive immune responses by stimulating the release of endogenous tumor-associated neoantigens and adjuvants, acting as an in situ vaccine. The dose of radiation as well as the intrinsic radiosensitivity of the tumor and the extent to which it exhibits tumor-specific neoantigens impact the optimal release of endogenous tumor-associated antigens and adjuvants. DC phagocytosis of antigens and exposure of adjuvants results in their activation and migration to the draining lymph node resulting in the activation of tumor-specific T cell responses [51,52]. On the contrary, macrophage phagocytosis of dying cancer cells [53] and inhibitory pathways that limit the effect of endogenous adjuvants [20] can limit DC activation and the associated activation of tumor-specific T cell responses. Thus, DCs are an essential link bridging the innate and adaptive immune responses associated with radiotherapy.

Radiotherapy can promote immunogenic cell death and the simultaneous release of adjuvants to induce a DC-mediated anti-tumor immune response. The early response is mediated by the secretion of inflammasome-related cytokines (tumor necrosis factor (TNF), IL-6, IL-1b, IFNs) and the production of reactive oxygen species to induce a pro-inflammatory microenvironment. This is followed by immunostimulatory DAMPs secreted by dying tumor cells such as passive release of HMGB1, calreticulin surface translocation, and ATP release that act on TLRs of DCs to promote their maturation [14]. Mature DCs increase expression of costimulatory molecules B7-1 and B7-2 that bind to CD28 on the T cell surface with the simultaneous engagement of peptide-MHC complex to promote an effector and memory T cell response [54]. Further, radiation-induced DAMPs such as double-stranded DNA can promote the release of mediators such as type I IFN that activate DCs via cGAS-STING pathway to promote CD8^+^ T cell activation [12,55,56,57]. Thus, radiation-induced ICD followed by the release of antigens and adjuvants can promote DC activation and CD8^+^ T cell-mediated adaptive immune responses.

**Figure 2:**
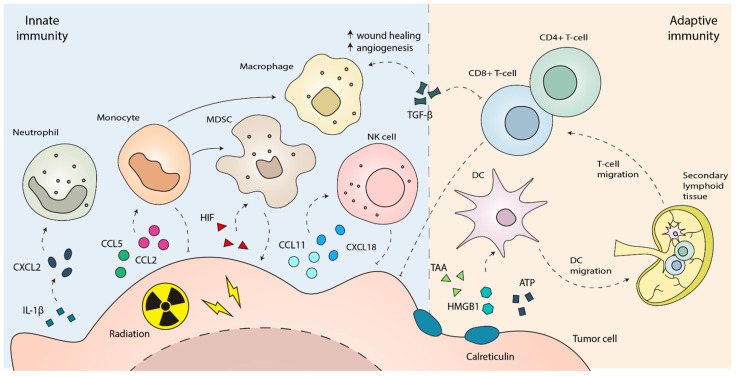
Radiation shapes the tumor microenvironment (TME) by activating both innate and adaptive immune responses. Diverse cell types within the TME are regulated by cytokines, including those derived from irradiated tumors, and collectively contribute to either pro-tumor or anti-tumor outcomes.

In contrast, the phagocytosis of dying tumor cells by macrophages can dampen DC-mediated adaptive immune responses [53]. Consequently, blocking macrophage phagocytosis by targeting Mertk or the CD47-SIRPa axis has been shown to enhance adaptive immune responses following radiotherapy [49,53,58]. Additionally, radioresistant tumors develop mechanisms to curb DC-mediated adaptive immune responses by the secretion of inhibitory molecules such as prostaglandin E2 (PGE2) or caspase-3, which recruits macrophages that promote a wound-healing response [59,60]. These tumors may also secrete growth factors such as vascular endothelial growth factor (VEGF), TGF-β or granulocyte-macrophage colony-stimulating factor (GM-CSF) that are involved in tissue homeostasis and immunosuppression [61]. Tumor-derived GM-CSF drives the accumulation of regulatory T-cells (Tregs) in the TME through generation of monocyte-derived DCs, and GM-CSF may therefore be a therapeutic target alongside radiotherapy [28]. In addition, inhibition of TGF-β can promote CD8^+^ T cell-mediated control of tumors following radiotherapy [62,63]. Other metabolic features of tumors such as IDO-1 can also limit CD8^+^ T cell adaptive responses while promoting the recruitment of immunosuppressive Tregs or MDSCs [11]. Given the critical role of adaptive immune responses to radiotherapy, combination therapies targeting Tregs or exhausted CD8^+^ T cells are an area of growing interest in radioimmunotherapy with the potential to improve treatment outcomes [64,65]. In all, the activation of both the innate and adaptive immune system through targeted irradiation dictates the overall immune response and influences clinical outcomes (Figure 2).

## 5. Systemic and Off-Target Immunomodulation by Radiotherapy

**Figure 3:**
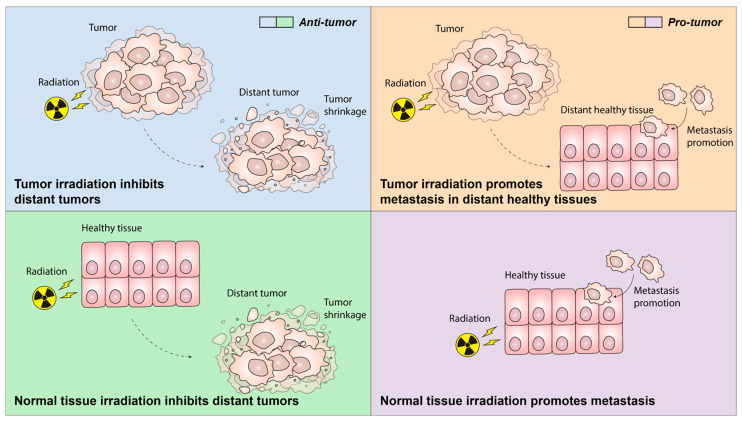
Systemic and off-target effects of radiation have been reported but remain incompletely understood. Localized tumor irradiation can trigger the abscopal effect, leading to inhibition of distant tumors, but it has also been shown to promote metastasis in normal tissue. Similarly, irradiation of non-tumor tissues may enhance metastatic spread, but has also been observed to suppress growth of distant tumors.

While the local effects of radiotherapy are more well-established, the systemic and off-target effects of radiotherapy on cancer and metastasis remain less understood and warrant further study (Figure 3). Radiotherapy induces systemic immune response that can affect tumor growth and metastasis in distant, non-irradiated tissues, a term known as abscopal effect. This phenomenon was first described in 1953, when RH Mole observed a normal tissue reaction at distant sites not directly subjected to irradiation [66]. In the clinic, anti-tumor abscopal effects remain rare [67,68]. While radiation can induce anti-tumor immune responses at primary sites, it also has the potential to enhance the metastatic potential of non-irradiated tissues through the modulation of systemic immune responses. In addition, the unintended exposure of normal tissue within the irradiated target volume can trigger systemic responses that may influence anti-tumor responses at these distant sites. Therefore, it is necessary to consider both the systemic and off-target effects of radiation on tumor and normal tissues when evaluating the holistic response generated by radiotherapy.

The critical role of the adaptive immune system in mediating the abscopal effect was initially recognized by Demaria and colleagues in 2004 [69], when they demonstrated that stimulation of DCs with FMS-like tyrosine kinase 3 ligand (FLT3L) in combination with radiation delayed the growth of secondary non-irradiated tumors. Crucially, this effect was not observed in immunodeficient mice, confirming the requirement for an intact immune system. Subsequent studies emphasized the importance of DC-mediated priming of CD8^+^ T-cells in supporting the abscopal effect [70], further highlighting the importance of the adaptive immune system in this process. In patients with metastatic solid tumors, the use of GM-CSF in combination with local radiotherapy to stimulate DC and adaptive immune response elicited abscopal responses in some individuals [71]. Given the central role of the adaptive immune system in mediating abscopal responses, attempts have been made to enhance this response by combining radiotherapy with immune checkpoint blockade (ICB), and these have been extensively reviewed elsewhere [72,73,74]. However, the efficacy of such approaches remains limited due to immunosuppression and insufficient activation of adaptive immunity. Indeed, the exposure by radiation of immunogenic mutations to the immune system is thought to support abscopal responses [65]. In addition, immunogenic tumors are more likely to drive an abscopal response due to their ability to stimulate systemic immune activation, whereas non-immunogenic tumors typically fail to elicit such responses [21].

Innate immune cells may also play a crucial role in potentiating abscopal responses. Macrophages have recently been demonstrated in preclinical studies to be an attractive target for abscopal responses. A recent study highlighted that the combination of radiotherapy alongside blocking of the “don’t eat me” signal CD47 promotes abscopal responses independent of CD8^+^ T-cells in a macrophage-dependent manner [49]. Interestingly, another recent study found that the simultaneous blockade of PD-L1 and CD47 with radiotherapy promoted the abscopal effect mediated by improving macrophage phagocytosis of damaged tumor cells, as well as CD8^+^ T cell activation [58]. Further research is needed to optimize combination therapies that effectively activate both the innate and adaptive immune system to maximize therapeutic benefits, both in terms of local and abscopal effects.

Apart from promoting anti-tumor responses in distant sites through the abscopal effect, irradiation has been associated with systemic immune changes that facilitate metastasis in distant tissues. The irradiation of breast tumors in mice resulted in increased lung metastasis, which is attributed to mesenchymal stromal cell-derived CCL5 recruiting macrophages to the lung to support lung metastasis [75]. In addition, a recent study highlighted that local tumor irradiation drove PD-L1 expression in myeloid cells, while promoting MDSC accumulation in the lungs that promoted lung metastasis [76]. More recently, it has also been demonstrated that radiotherapy induces the expression of amphiregulin (AREG) in tumor cells, which facilitates metastasis through reprogramming myeloid cells towards an immunosuppressive phenotype [77]. In all, targeted radiation therapy drives systemic changes in the immune landscape of distant non-irradiated tumors and tissue, which could support anti-tumor responses against distant tumors or could influence the pre-metastatic immune microenvironment to support metastasis.

As previously discussed, irradiation of normal tissues can lead to the release of DAMPs and the recruitment of immune cells to these tissues. Consequently, immune cells recruited to irradiated tissue often can contribute to radiation-induced toxicity. One of the most well-studied examples is radiation-induced lung fibrosis, where fibrotic progression is driven by chronic wound-healing Type-2 responses involving the accumulation of Tregs, T_H_2 cells and M2-polarised macrophages [72,78,79]. Notably, preclinical studies suggest that the metastatic potential of irradiated normal tissues can be modulated by the inflammatory environment post-irradiation. In mice, targeted irradiation of normal lung tissue promoted the recruitment of neutrophils, leading to downstream activation of Notch signaling in epithelial cells that enhances metastasis [80]. In another study, irradiation of normal tissue stroma in immunodeficient mice induced CCL3, CCL4 and CCL5 secretion, attracting macrophages that facilitated tumor cell recruitment [81]. This effect was abrogated in the presence of CD8^+^ T-cells. The significance of normal tissue radiation in modulating the pre-metastatic niche in humans remains to be fully characterized.

While the systemic immune effects of normal tissue irradiation on cancer are poorly understood, irradiation of the gut appears to play a key role in regulating systemic anti-tumor responses. An early study demonstrated that pre-irradiation of the abdomen led to the inhibition of lung metastasis [82], while a later study by the same authors attributed this effect to the irradiation of the caecum [83]. Notably, the later study also found that inhibition of lung metastasis was not observed in germ-free mice, suggesting the importance of the gut microbiota in mediating systemic responses. Indeed, it is now increasingly recognized that dysbiosis of the gut microbiome influences cancer progression and survival in lung cancer patients [84,85]. A recent study hints at a possible mechanistic insight into this process. It was observed that low dose gut irradiation combined with PD-L1 blockade improved survival in advanced cancer patients [86]. This was attributed to the immunostimulatory effects of the *Christensenellaceae* species, which facilitated the migration of mregDC (PD-L1^+^CD40^+^ DCs) to the tumor-draining lymph nodes to prime anti-tumor CD8^+^ T-cell responses [86]. Therefore, low dose irradiation of the gut may enhance systemic anti-tumor immunity. Conversely, higher doses of irradiation to the gut appear to have the opposite effect. In another study, conformal irradiation of murine tumors promoted recruitment of IFN-I-producing monocytes that promoted anti-tumor CD8^+^ T-cell responses, whereas non-conformal radiotherapy resulting in damage to the gut limited the recruitment of these monocytes [27]. Therefore, the beneficial effect of gut irradiation appears to be dose dependent, and further research is needed to understand the influence of radiation to different tissue types on systemic immune response against tumors and metastasis.

## 6. Future Perspectives

Our growing understanding of the immunomodulatory effects of radiotherapy has been instrumental in supporting the clinical basis of combining radiotherapy with immunotherapy. Indeed, clinical trials combining radiotherapy and immunotherapy have steadily increased over the years, underscoring the need to better understand radiation-induced immune responses to maximize the success of these trials. Much of the translational effort in this area has focused on integrating ICB to maximize T-cell-mediated anti-tumor response, with anti-programmed cell death protein 1 (PD-1) and anti-PD-L1 therapies being the most widely explored. A key rationale for this combination is that radiotherapy can induce PD-L1 upregulation on tumor cells [87,88], effectively dampening T-cell activity. Blocking PD-1 or PD-L1 releases this checkpoint to overcome adaptive immune resistance and enhance anti-tumor immunity. In line with this strategy, over one-third of ongoing or completed clinical trials integrating radiotherapy with immunotherapy utilize PD-1 or PD-L1–targeted strategies (data from ClinicalTrials.gov). However, it is crucial to acknowledge that anti-tumor immunity is not limited to adaptive mechanisms, and fully realizing the therapeutic potential of radiotherapy will depend on effectively harnessing the innate immune response [89]. Further efforts could be considered to target innate immune responses. An emerging area of opportunity lies in harnessing innate immunity to amplify systemic responses and the abscopal effect. Of note, macrophages have recently emerged as potential regulators of the abscopal effect [49,58], suggesting that macrophage-targeting strategies, such as CD47 blockade, could be explored in combination with radiotherapy to enhance innate immunity and strength the systemic anti-tumor response.

A major challenge in the field is the translation of robust abscopal effects produced in preclinical models in the clinical setting, where reports of distant tumor regression through localized radiotherapy remain rare. A key reason is the radiation dose and fractionation regimen, which often differ substantially between preclinical studies and clinical practice. In addition, within the local irradiated TME, different cell types exhibit distinct radiosensitivity [90] and may be differentially activated depending on dose administered [48]. Even less is known about how differential radiation dosing regulates the systemic immunomodulatory response. Although some studies have examined dose-dependent influences on the abscopal response in both preclinical and clinical settings [91,92], our understanding of the mechanisms underlying these dose effects remains limited and warrant further study to translate our preclinical knowledge to the clinic.

Another key factor may be the limited consideration of the role of normal tissue exposure on systemic immune responses generated in the TME, which is especially important to consider in the treatment of tumors where irradiation of adjacent normal tissue is unavoidable in the clinical setting. Recent studies have identified the gut as a promising target for irradiation to promote systemic anti-tumor responses, likely through the immune-microbiota axis [93]. Cross-communication between the gut and lung immune systems has been reported in various pathological contexts, although whether radiotherapy-induced modulation of lung microbiota affects systemic anti-tumor immunity remains unknown [94,95,96]. Therefore, understanding the microbiota’s influence on the abscopal response may provide valuable insights for developing new therapies.

Parallel research efforts aimed at minimizing radiation damage to normal tissues including application of radioprotectors and radiomitigators [97,98] and the use of ultra-high dose rate (FLASH) radiotherapy [99] may have additional influence on the incidence of abscopal effects. In all, further studies are needed to elucidate mechanisms bridging the gaps between preclinical and clinical observations of systemic radiation effects.

## 7. Conclusions

Overall, the systemic and off-target immunological effects of radiotherapy represent an important and evolving area of research. Our current understanding of immune responses to radiotherapy has been primarily shaped by our understanding of the local effects of targeted tumor irradiation, including how these radiation-induced signals drive immune recruitment and activation in the tumor microenvironment. However, increasing attention is now directed towards understanding the systemic off-target effects of localized radiotherapy, such as how irradiation of a single tumor influences distant, non-irradiated lesions (the abscopal effect) or alters immune homeostasis in other tissues. A holistic and thorough understanding of the systemic immunological consequences in both tumor and normal tissue will be crucial for guiding future strategies to enhance treatment outcomes and optimize combination therapies involving radiotherapy.

## Abbreviations

The following abbreviations are used in this manuscript:

AREG	Amphiregulin
ATP	Adenosine triphosphate
ATR	Ataxia-Telangiectasia Mutated and Rad3-Related
cGAS	Cyclic GMP-AMP synthase
CSF1	Colony stimulating factor 1
DAMPs	Damage-associated molecular patterns
DCs	Dendritic cells
dsDNA	Double stranded-DNA
FAS	Fas cell surface death receptor
FLT3L	FMS-like tyrosine kinase 3 ligand
GM-CSF	Granulocyte-macrophage colony-stimulating factor
HIF	Hypoxia-inducible factor
HMGB1	High mobility group box 1 protein
ICB	Immune checkpoint blockade
ICD	Immunogenic cell death
IDO-1	Indoleamine 2,3-dioxygenase 1
IFN-I	Type 1 interferon
MDSCs	Myeloid-derived suppressor cells
MHCI	Major histocompatibility complex I
NK	Natural killer
NLRP3	NOD-, LRR- and pyrin domain-containing protein 3
PD-1	Programmed cell death protein 1
PD-L1	Programmed death-ligand 1
PGE	Prostaglandin E2
RIG1	Retinoic acid-inducible gene I
SIRPα	Signal regulatory protein α
STING	Stimulator of interferon genes
TGFβ	Transforming growth factor β
TLR4	Toll-like receptor 4
TME	Tumor microenvironment
TNF	Tumor necrosis factor
Treg	Regulatory T-cell
VEGF	Vascular endothelial growth factor
